# Supplementary material for: Effects of Typical Antimicrobials on Growth Performance, Morphology and Antimicrobial Residues of Mung Bean Sprouts
Source: Antibiotics (Basel). 2022 Jun 15;11(6):807. doi: 10.3390/antibiotics11060807 (PMC9219749; doi:10.3390/antibiotics11060807)
Supplement: Supplementary file 1 [file antibiotics-11-00807-s001.zip › antibiotics-1738611-supplementary.pdf]

Supplementary materials

1. **Table S1** Positive ion A gradient elution conditions
2. **Table S2** Positive ion B gradient elution conditions
3. **Table S3** Negative ion gradient elution conditions
4. **Table S4** Mass spectrometry conditions in MRM mode
5. **Figure S1** Correlation between production of mung bean sprouts and OD450 in circulating water in 96 h

Table S1 Positive ion A gradient elution conditions

step	Total time (min)	A (%)	B (%)	flow rate
1	0.00	90	3	0.4
2	1.00	90	3	0.4
3	1.10	60	15	0.4
4	9.50	40	75	0.4
5	9.60	10	95	0.4
6	11.50	10	95	0.4
7	11.60	90	3	0.4
8	13.00	97	3	0.4

Table S2 Positive ion B gradient elution conditions

step	Total time (min)	A (%)	B (%)	flow rate
1	0.00	90	10	0.3
2	0.50	90	10	0.3
3	3.00	60	40	0.3
4	6.00	40	60	0.3
5	6.50	10	90	0.3
6	8.00	10	90	0.3
7	8.20	90	10	0.3

Table S3 Negative ion gradient elution conditions

step	Total time (min)	A (%)	B (%)	flow rate
1	0.00	80	20	0.5
2	0.50	80	20	0.5
3	2.80	2	98	0.5
4	4.00	2	98	0.5
5	4.10	80	20	0.5
6	6.00	80	20	0.5

Table S4 Mass spectrometry conditions in MRM mode

condition	positive ions	negative ions
electrospray voltage	5500	-4500
curtain air pressure	30 psi	30 psi
ion source atomizer temperature	600°C	600°C
atomizing gas pressure	50 psi	50 psi
auxiliary gas pressure	60 psi	60 psi
Ihe	on	on

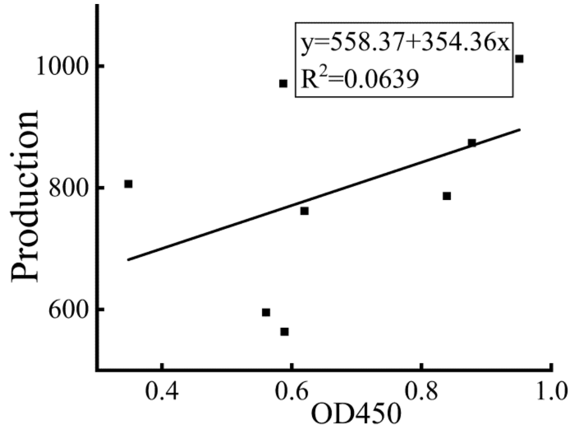

Figure S1. Correlation between production of mung bean sprouts and OD450 in circulating water in 96h.
